# Identification and characterization of a carnitine transporter in *Acinetobacter baumannii*

**DOI:** 10.1002/mbo3.752

**Published:** 2018-10-14

**Authors:** Jennifer Breisch, Izabela Waclawska, Beate Averhoff

**Affiliations:** ^1^ Department of Molecular Microbiology & Bioenergetics, Institute of Molecular Biosciences Goethe‐University Frankfurt am Main Frankfurt Germany; ^2^ Institute of Biophysics & Biophysical Chemistry University Regensburg Regensburg Germany

**Keywords:** *Acinetobacter baumannii*, betaine/choline/carnitine transporter, carnitine metabolism, human host adaptation, human pathogen

## Abstract

The opportunistic pathogen *Acinetobacter baumannii* is able to grow on carnitine. The genes encoding the pathway for carnitine degradation to the intermediate malic acid are known but the transporter mediating carnitine uptake remained to be identified. The open reading frame HMPREF0010_01347 (*aci01347)* of *Acinetobacter baumannii* is annotated as a gene encoding a potential transporter of the betaine/choline/carnitine transporter (BCCT) family. To study the physiological function of Aci01347, the gene was deleted from *A. baumannii* ATCC 19606. The mutant was no longer able to grow on carnitine as sole carbon and energy source demonstrating the importance of this transporter for carnitine metabolism. Aci01347 was produced in *Escherichia coli* MKH13, a strain devoid of any compatible solute transporter, and the recombinant *E. coli* MKH13 strain was found to take up carnitine in an energy‐dependent fashion. Aci01347 also transported choline, a compound known to be accumulated under osmotic stress. Choline transport was osmolarity‐independent which is consistent with the absence of an extended C‐terminus found in osmo‐activated BCCT. We propose that the Aci01347 is the carnitine transporter mediating the first step in the growth of *A. baumannii* on carnitine.

## 1 INTRODUCTION

The genus *Acinetobacter* comprises Gram‐negative proteobacteria known for their extraordinary metabolic diversity (Baumann, Doudoroff, & Stanier, 1968). Many of the species are environmental isolates, whereas some are part of the human microbiome or even opportunistic pathogens such as *Acinetobacter baumannii* (Berlau, Aucken, Houang, & Pitt, 1999; Peleg, Seifert, & Paterson, 2008). *Acinetobacter* species are known to degrade aromatic compounds via the β‐ketoadipate pathway (Ornston & Parke, 1977) but can also grow on sugars, acids such as succinate or alcohols such as ethanol (Abbott, Laskin, & Mccoy, 1973; Bouvet & Grimont, 1986; Young, Parke, & Ornston, 2005). In addition, quaternary ammonium compounds are also known to play pivotal roles in the physiology of *Acinetobacter* (Kleber, Seim, Aurich, & Strack, 1977).

Choline, 2‐hydroxyethyl trimethylammonium chloride, a building block of lipids present in animals and plants, is ubiquitous in the environment (Bernhard et al., 2001) and is taken up by *Acinetobacter baylyi* and oxidized *via* glycine betaine aldehyde to glycine betaine by the action of a choline dehydrogenase (BetA) and a glycine betaine aldehyde dehydrogenase (BetB; Sand, Stahl, Waclawska, Ziegler, & Averhoff, 2014,; Scholz, Stahl, Berardinis, Müller, & Averhoff, 2016). This pathway does not serve as sole energy conserving pathway, choline is only co‐metabolized, but choline oxidation to glycine betaine has an energetic benefit for the cell. More important, the product of the reaction sequence, glycine betaine, is a known osmoprotectant. Therefore, the expression of the choline transporter and the choline oxidation pathway genes is regulated by the transcriptional regulator BetI (Sand et al., 2014, Scholz et al., 2016) via the choline and salt concentration of the environment. Thus, induction of choline uptake and oxidation is induced by the presence of choline in the medium and in addition, by high salt (Sand et al., 2014).

Glycine betaine is used by *A. baylyi* (Sand et al., 2011) and *A. baumannii (*Zeidler et al., 2018 *)* as compatible solute. It cannot be synthesized de novo but from the precursor choline, as outlined above. In addition, both species can take up glycine betaine from the medium (Sand et al., 2011; Zeidler et al., 2018) and uptake is preferred over synthesis from choline for energetic reasons (Oren, 1999, Sand et al., 2014). In contrast to, for example, *Pseudomonas aeruginosa* (Lisa, Garrido, & Domenech, 1983), glycine betaine is not further metabolized. When the osmolarity in the ecosystem decreases, glycine betaine is expelled by yet to be identified proteins.

Another quaternary amine found in many soil ecosystems but most abundantly associated with animals is carnitine. It is found in animal tissues, particularly muscle, at high concentrations where it contributes to fatty acid transport into mitochondria (Wang, Meadows, & Longo, 2000). Carnitine can be metabolized by many bacteria and also serves as osmoprotectant in different Gram‐negative and Gram‐positive bacteria (Jebbar, Champion, Blanco, & Bonnassie, 1998; Jung, Jung, & Kleber, 1990; Kappes & Bremer, 1998; Kets, Galinski, Debont, & a. M., 1994; Kleber, 1997; Lucchesi, Lisa, Casale, & Domenech, 1995; Meadows & Wargo, 2015). Carnitine has not been reported as a compatible solute in the genus *Acinetobacter*. Under anaerobic conditions, *Escherichia coli* can oxidize carnitine to γ‐butyrobetaine that is expelled from the cell by a carnitine:γ‐butyrobetaine antiporter, CaiT (Schulze, Köster, Geldmacher, Terwisscha Van Scheltinga, & Kühlbrandt, 2010). In contrast, *Acinetobacter calcoaceticus* uses carnitine as sole carbon and energy source. The first step is the splitting of the C‐N bond in carnitine leading to the release of trimethylamine and malic acid semialdehyde. Subsequent oxidation gives malic acid, a central intermediate of the citric acid cycle that is oxidized to CO_2_ (Kleber et al., 1977; Seim, Löster, & Kleber, 1982). Recently, mining for carnitine‐degrading enzymes in reference genomes of a Human Microbiome Project (HMP) led to the detection of a putative carnitine utilization gene cluster in *Acinetobacter* ssp. (Zhu et al., 2014). Mutant studies of *A. baumannii* ATCC 19606 led to the identification of the carnitine oxygenase genes *cntA* and *cntB*, required for the oxidation of carnitine to malic acid (Zhu et al., 2014). The transporter catalyzing carnitine uptake had not been described yet but close to *cntA/B* is a gene (*aci01347*) encoding a potential transporter of the betaine/choline/carnitine transporter (BCCT) family. Indeed, this gene was suggested by Zhu et al. to encode a carnitine transporter. Herein, we provide genetic and biochemical evidence that Aci01347 of *A. baumannii* is a carnitine transporter essential for growth on carnitine.

## 2 MATERIALS AND METHODS

### 2.1 Bacterial strains and growth conditions

*Escherichia coli* MKH13 strains were grown in LB medium (Bertani, 1951) at 37°C with 100 µg/ml ampicillin. *A. baumannii* strain ATCC 19606 was grown at 37°C in LB medium (Bertani, 1951) or in mineral medium (MM) that consists of 50 mM phosphate buffer, pH 6.8, and mineral solution (composition per liter: 1 g NH_4_Cl, 580 mg MgSO_4_ × 7 H_2_O, 100 mg KNO_3_, 67 mg CaCl_2_ × 2 H_2_O, 2 mg (NH_4_)_6_Mo_7_O_24_ × 4 H_2_O, 1 ml SL9 (per liter: 12.8 g Titriplex, 2 g FeSO_4_ × 7 H_2_O, 190 mg CoCl_2_ × 6 H_2_O, 122 mg MnCl_2_ × 4 H_2_O, 70 mg ZnCl_2_, 36 mg MoNa_2_O_4_ × 2 H_2_O, 24 mg NiCl_2_ × 6 H_2_O, 6 mg H_3_BO_3_, 2 mg CuCl_2_ × H_2_O; pH 6.5) (Tschech & Pfennig, 1984) with 20 mM sodium acetate or carnitine as carbon source. The role of choline and carnitine as compatible solutes was addressed by growth experiments in mineral medium with 300 mM NaCl in the presence of 1 mM choline and carnitine, respectively. Kanamycin (50 µg/ml) or gentamicin (100 µg/ml) was added from stock solutions when appropriate. Growth was monitored by measurement of the optical density at 600 nm. The growth experiments were repeated 3 times, and one representative experiment is shown. Growth curves were fitted manually.

### 2.2 Cloning of *aci01347* in *E. coli* MKH13

The *aci01347* gene was cloned with a histidine tag into the vector pBAD/HisA into the MCS (*Sac*I and *Eco*RI). The histidine tag (his_6_) was added at the N‐terminus. The primers pBAD/HisA_*aci01347*_fwd and pBAD/HisA_*aci01347*_rev (Supporting Information Table S1) were used to amplify the *aci01347* gene from the genome of *A. baumannii* ATCC 19606. The plasmid was then transferred into *E. coli* MKH13, which is devoid of all compatible solute transporter.

### 2.3 [^14^C]‐choline and [^14^C]‐glycine betaine uptake in *E. coli* MKH13

*Escherichia coli* MKH13 carrying the plasmid pBAD/HisA_*aci01347* and a control strain carrying pBAD/HisA were cultivated at 37°C in LB medium containing ampicillin (100 µg/ml). Expression of the transporter was induced at an OD_600_ of 0.6–0.8 with 0.02% arabinose. The cells were harvested 2.5 hr after induction and washed in 0.5 volume KPi‐buffer pH 7.5 (25 mM) with 100 mM NaCl. Cells were resuspended in the same buffer containing 30 mM glucose to an OD_600_ of 3. Different osmolalities of the KPi‐buffer were adjusted with KCl. For compatible solute uptake studies, cell suspensions were diluted 1:1 with the adjusted KPi‐buffer, and after 3 min of incubation at 37°C, the assay was started by adding 500 µM [^14^C]‐substrate (1 µCi). Samples (200 µl) were taken at time points indicated. Cells were separated from the medium by filtration using mixed cellulose nitrate filters (pore size 0.45 µm, Sartorius Stedim, Göttingen, Germany) and washed with 20 volumes 0.6 M KPi‐buffer. The filters were dried and then dissolved in 4 ml scintillation fluid (Rotizint^R^ eco plus; Carl‐Roth GmbH, Karlsruhe, Germany); the radioactivity was determined by a liquid scintillation counter.

### 2.4 Markerless mutagenesis of *aci01347*

To generate a markerless *A. baumannii* Δ*aci01347* (HMPREF0010_01347) mutant, the 1500 bp up‐ and downstream regions of *aci01347* were amplified from *A. baumannii* ATCC 19606 genomic DNA using the primer pairs *aci01347*_up_fwd + *aci01347*_up_rev (Supporting Information Table S1) and *aci01347*_down_fwd + *aci01347*_down_rev (Supporting Information Table S1) and inserted in *Not*I and *Pst*I digested pBIISK_*sacB/kanR*. The resulting plasmid pBIISK_*sacB/kanR*_*aci01347_*updown was purified and transformed in electrocompetent *A. baumannii* ATCC 19606 cells. To generate electrocompetent cells, *A. baumannii* ATCC 19606 was grown in LB medium, harvested at OD_600_ = 0.45, washed four times in H_2_O_MQ_, and resuspended in 10% glycerol. Electroporation was performed at 2.5 kV, 200 Ω, and 25 µF. Transformants were selected on LB‐agar containing 50 µg/ml kanamycin. The integration of the vector into the *aci01347* loci was verified by PCR using the primer *aci01347*_ctr_fwd + *aci01347*_down_rev and *aci01347*_up_fwd + *aci01347*_ctr_rev (Supporting Information Table S1). Integrants were grown overnight in LB containing 10% sucrose, plated on LB‐agar containing 10% sucrose, and single colonies were analyzed by replica plating onto LB/kanamycin agar with respect to kanamycin sensitivity. The deletion of the *aci01347* gene was verified by PCR using the primers *aci01347*_ctr_fwd + *aci01347*_ctr_rev (Supporting Information Table S1).

### 2.5 Complementation of *aci01347* mutants

For complementation studies, the vector pVRL1 was used (Lucidi et al., 2018). The *aci01347* gene and the promotor region were amplified from genomic DNA using the primer pairs *aci01347*_compl_fwd + *aci01347*_compl_rev and *aci01347*_promotor_fwd + *aci01347*_promotor_rev (Supporting Information Table S1) and inserted in *Pst*I and *Not*I digested pVRL1. The resulting plasmid pVRL1_*aci01347* and the pVLR1 vector control were transformed into *A. baumannii* Δ*aci01347* by electrotransformation. Transformants were selected on LB‐agar containing 100 µg/ml gentamicin. *A. baumannii* Δ*aci01347* transformants carrying either pVRL1_*aci01347* or the vector pVLR1 were grown in MM with 20 mM carnitine as carbon source ensuing from a preculture with 20 mM sodium acetate as carbon source.

## 3 RESULTS

### 3.1 Characterization of the potential BCCT gene *aci01347* and its deduced product

Inspection of the *A. baumannii* ATCC 19606 genome revealed the presence of a gene, *aci01347,* that encodes a potential, secondary active betaine/choline/carnitine transporter and is highly conserved in the *A. baumannii* strains (data not shown).

**Figure 1:**
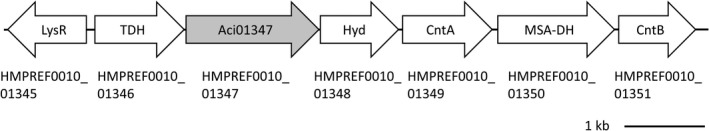
Genetic organization of the *aci01347* locus of *Acinetobacter baumannii*. The potential regulator of the genes essential for carnitine oxidation is labeled with LysR, TDH is a predicted tartrate dehydrogenase, CntA/B was found to encode an unusual Rieske‐type oxygenase (Zhu et al., 2014), MSA‐DH is a potential malate semialdehyde dehydrogenase, and Aci01347 is a potential BCCT

The gene is physically located in a gene cluster (Figure 1) producing proteins involved in carnitine metabolism (21 bp downstream of the malate dehydrogenase and 12 bp upstream of the acylcarnitine hydrolase; Zhu et al., 2014). *Aci01347* encodes a 551‐amino acid protein with a molecular mass of 60.9 kDa with 12 predicted transmembrane helices (predicted with the software TMPres2D and the server HMMTOP; Tusnady & Simon, 2001), connected by short loops. Aci01347 exhibits the highest similarity (33% amino acid identity and 68% similarity) to the choline transporter CudT in *Staphylococcus aureus*. Comparison of Aci01347 with the osmolarity‐independent choline transporter BetT1 and the osmo‐activated choline transporter BetT2 of *A. baylyi* revealed low identities of only 28% and 25%, respectively.

### 3.2 Deletion of *aci01347* abolishes growth on carnitine

**Figure 2:**
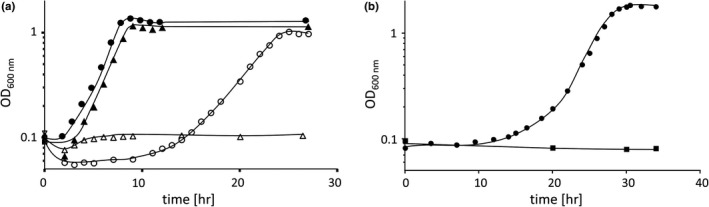
(a) Growth of *Acinetobacter baumannii* ATCC19606 wild‐type (●/○) and Δ*aci01347* mutant (▲/Δ) in mineral medium on either acetate (20 mM; closed symbols) or carnitine (20 mM; open symbols) as sole carbon and energy source. (b) The *Δaci01347‐*mutant was transformed with either pVRL1_*aci01347* (●) or vector pVRL1 (■), and growth of both strains was followed in mineral medium with 20 mM carnitine as sole carbon source

To address the role of Aci01347 in *A. baumannii* ATCC 19606, the gene was deleted from the chromosome *via* single homologous recombination and segregation of the plasmid using *sacB,* yielding the markerless deletion mutant *Δaci01347* (Stahl, Bergmann, Göttig, Ebersberger, & Averhoff, 2015)*.* The deletion mutant was verified by DNA sequencing. The *Δaci0134* mutant was completely defect in growth on carnitine, whereas growth on acetate was not affected (Figure 2a). To validate the role of *aci01347* in carnitine metabolism, the *Δaci01347* mutant was complemented with plasmid pVRL1_*aci01347* containing the *aci01347* gene under the control of the native promotor. The complemented mutant grew with carnitine as sole carbon and energy source, whereas the mutant with the empty vector pVRL1 did not (Figure 2b). This together with the similarities of Aci01347 to BCCT strongly suggests that *aci01347* encodes a carnitine transporter. Moreover, the complete loss of the ability to use carnitine as sole carbon and energy source leads to the conclusion that Aci01347 is essential for growth on carnitine.

In previous studies, we analyzed the induction of two distinct choline transporter in *A. baylyi* and found that they are maximally induced in the presence of choline and high salt (Sand et al., 2014). High salt also induces the synthesis of glycine betaine transporter. To address the question whether other potential choline transporter or glycine betaine transporter encoded by *A. baumannii* are also able to mediate carnitine uptake, the *A. baumannii Δaci01347* mutant was grown in mineral medium with carnitine as carbon source in the presence of 1 mM choline and 300 mM NaCl. Still no growth was observed (data not shown) indicating that Aci01347 is the only carnitine transporter in *A. baumannii*.

### 3.3 Aci01347 is a secondary active transporter for quaternary ammonium salts

To study the biochemical function of Aci01347, the encoding gene was cloned into the expression vector pBAD/HisA, and the plasmid was transformed into *E. coli* MKH13. This strain of *E. coli* is devoid of any transporter for compatible solutes and thus an ideal host to study the function of heterologously produced BCCT (Haardt, Kempf, Faatz, & Bremer, 1995). Transformants were grown in LB medium, washed, and resuspended in KPi‐buffer pH 7.5 with 100 mM NaCl and 30 mM glucose. The cell suspensions were used for the transport experiments.

**Figure 3:**
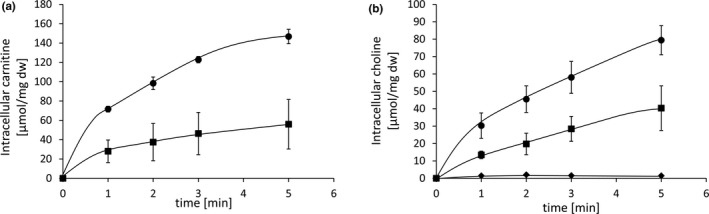
[^14^C]‐carnitine uptake (a) and [^14^C]‐choline uptake (b) by *Escherichia coli* MKH13‐*aci01347*. Uptake of [^14^C]‐carnitine and [^14^C]‐choline was measured in KPi‐buffer (25 mM, pH 7,5) at external osmolality of 0.2 osmol/kg in *E. coli* MKH13 cells expressing Aci01347 of *Acinetobacter baumannii.* Uptake was started by adding saturating concentrations of 500 µM [^14^C]‐carnitine or [^14^C]‐choline (0.1 µCi) (●). The effect of the protonophore TCS was analyzed by [^14^C]‐carnitine and [^14^C]‐choline uptake studies after preincubation of the cells with 50 µM TCS (■). [^14^C]‐choline uptake was further measured in the presence of 50‐fold excess of carnitine (♦). Each value is the mean ± *SEM* of at least three independent measurements

To verify a function of Aci01347 in carnitine uptake, resting cells were incubated in the presence of 500 µM [^14^C]‐carnitine. As can be seen in Figure 3 A, cells accumulated [^14^C]‐carnitine with a rate of 27 µmol/min up to 147 µmol/mg dry weight, whereas *E. coli* MKH13 cells did not accumulate [^14^C]‐carnitine (data not shown). The presence of 50 µM of the protonophore TCS (3,3′,4′,5‐tetrachlorosalicylanilide) largely impaired [^14^C]‐carnitine uptake (Figure 3a), demonstrating that carnitine uptake is energy‐dependent.

To analyze a function of Aci01347 in choline uptake, radioactively labeled [^14^C]‐choline (Figure 3b) was added to the cells at a concentration of 500 µM. As can be seen in Figure 3b, cells also took up [^14^C]‐choline from the medium, albeit with a lower rate (19 µmol/min up to 98 µmol/mg dry weight). Transport again was energy‐dependent and inhibited by the protonophore TCS. These data demonstrate that Aci01347 translocates choline. Cross‐competition uptake assays were performed to analyze whether Aci01347 mediates the uptake of other compatible solutes as well. Therefore, cells were incubated in the presence of 10 µM [^14^C]‐labeled choline and unlabeled potential competitors such as proline, proline betaine, carnitine, ectoine, or glycine betaine were added to the transport assay mixtures at a concentration of 0.5 mM. The 50‐fold excess of proline, proline betaine, ectoine, or glycine betaine had no effect or very little effect on the choline uptake activity of *A. baumannii*. The choline uptake was reduced by 4.6%, 2.4%, 9.2%, and 14.5%, respectively. However, only addition of 50‐fold excess of carnitine led to a complete inhibition of [^14^C]‐choline transport (Figure 3b). This complete inhibition of choline uptake by carnitine implies that Aci01347 mediates the uptake of both, choline and carnitine, at least under these conditions.

### 3.4 Aci01347 is not activated by osmolarity

**Figure 4:**
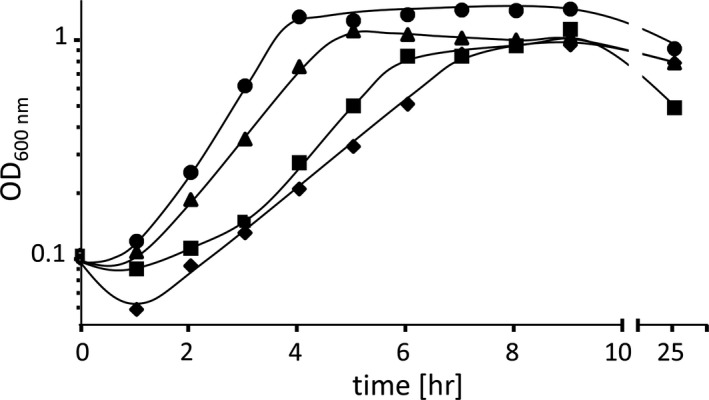
Protection of *Acinetobacter baumannii* against salt stress by choline. The cells were grown in MM (●), in the presence of 300 mM NaCl (■), in the presence of 300 mM NaCl and 1 mM choline (▲) or in the presence of 300 mM NaCl and 1 mM carnitine (♦)

**Figure 5:**
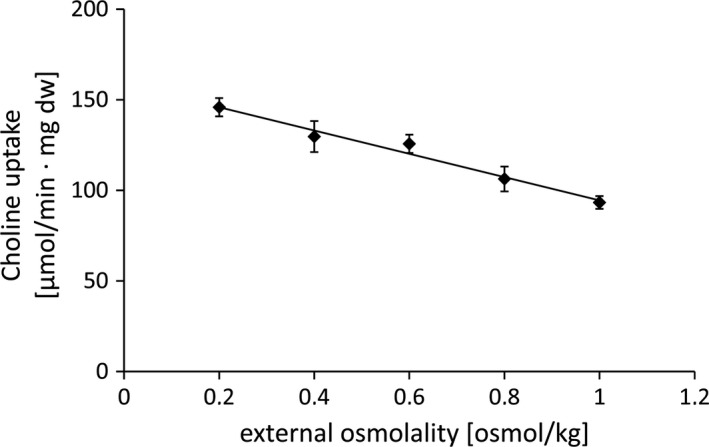
Osmostress‐independent choline uptake by *Escherichia coli* MKH13‐*aci01347*. Uptake of [^14^C]‐choline was measured at external osmolalities of 0.2, 0.4, 0.6, 0.8, and 1 osmol/kg and over 2.75 min. The uptake was started by the addition of 500 µM choline and 1 µCi [^14^C]‐choline (carrier‐free). Each value is the mean of at least three independent measurements

The finding that Aci01347‐mediated choline uptake raised the question whether Aci01347 is also involved in osmoadaptation. Indeed, the growth of *A. baumannii* at high salt was stimulated by choline (Figure 4). This led us to determine a possible osmoactivation of the protein. To this end, *E. coli* MKH13 pBAD/HisA_*aci01347* was washed with KPi‐buffer containing 0.2 osmol/kg and resuspended in buffer containing an external osmolality of 0.2 osmol/kg, 0.4 osmol/kg, 0.6 osmol/kg, 0.8 osmol/kg, or 1 osmol/kg. As can be seen in Figure 5, Aci01347‐mediated [^14^C]‐choline uptake activity was not activated by increasing osmolalities but rather decreased over an external osmolality range from 0.2 to 1 osmol/kg. This is consistent with the hypothesis that Aci01347 is not osmotically activated. In line with this hypothesis is the finding that carnitine did not stimulate growth of *A. baumannii* at high salt (Figure 4).

## 4 DISCUSSION

Analyses of the carnitine degradation of *A. baumannii* ATCC 19606 led to the identification of a two‐component Rieske‐type oxygenase/reductase (CntAB) essential for the metabolism of carnitine to TMA and malate semialdehyde (Zhu et al., 2014). The *cntA/B* genes were found to be associated with genes likely to be involved in C4 metabolism, transcriptional regulation, and transport. However, the function of genes potentially involved in carnitine uptake and metabolism has not been addressed yet. Here, we present the first functional characterization of the carnitine transporter in *A. baumannii*. We performed mutant studies and biochemical analyses and provide clear evidence that *aci01347* encodes a BCCT mediating the uptake of both, choline and carnitine. Furthermore, we found that Aci01347 is not activated by osmolarity. The uptake of both choline and carnitine by a classical member of the BCCT family is quite unique and without precedence. Carnitine transport and metabolism have already been thoroughly studied in different bacteria such as *Pseudomonas aeruginosa* and in members of the *Enterobacteriaceae* (Meadows & Wargo, 2015). In *P. aeruginosa,* carnitine is imported by an ABC transporter and can be used as sole carbon and nitrogen source but also plays a role in osmoprotection and virulence factor induction (Chen, Malek, Wargo, Hogan, & Beattie, 2010; Kleber, Schöpp, Sorger, Tauchert, & Aurich, 1967; Lucchesi et al., 1995; Wargo & Hogan, 2009). In *Enterobacteriaceae*, such as *E. coli*,* Salmonella typhimurium,* or *Proteus vulgaris,* it was found that under anaerobic conditions, when no other electron acceptor is present, carnitine is metabolized to γ‐butyrobetaine and carnitine uptake is mediated by a member of the BCCT family which functions as a substrate:product antiporter (Eichler, Bourgis, Buchet, Kleber, & Mandrand‐Berthelot, 1994; Jung et al., 2002). The carnitine transporter in *A. baumannii* differs from the carnitine transporter in *P. aeruginosa* and *E. coli*. Aci01347 is probably a proton:substrate symporter, as other choline transporter (Sand et al., 2014) and the first non carnitine:γ‐butyrobetaine transporter described. In silico structural analyses of the glycine betaine transporter BetP of *Corynebacterium glutamicum* led to the identification of two sodium‐binding sites. The first sodium‐binding site comprises of T246, T250, and F380, whereas the second sodium‐binding site is formed by M150, A147, F464, T467, and S468 (Khafizov et al., 2012). These sodium‐binding sites are not present in Aci01347, which corresponds with our suggestion that Aci01347 is probably a proton:substrate symporter.

**Figure 6:**
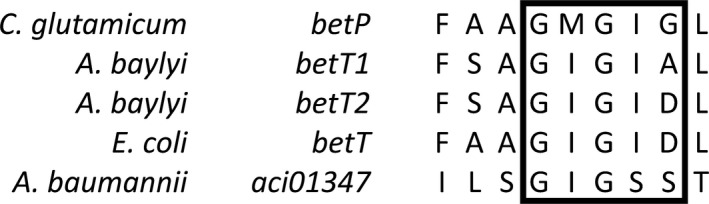
Multisequence alignment of the glycine motif in TM3 of BetP from *C. glutamicum*, BetT1 and BetT2 from *A*. *baylyi*, BetT from *E. coli,* and Aci01347 from *A. baumannii*, respectively

Aci01347 of different *A. baumannii* strains contains a conserved glycine‐rich segment in transmembrane helix three with the GMGIG motif which is typical for glycine betaine transporter and slightly different in choline‐specific transporter (GIGIA/D; Figure 6) but absent in the substrate:product antiporter CaiT of *E. coli* (Sand et al., 2014, Lamark et al., 1991). The charged residue in this motif is suggested to be crucial for substrate co‐ordination for proton‐coupled transporter of the BCCT family (Ziegler, Bremer, & Krämer, 2010). A serine is present in position four of the glycine motif in Aci01347 instead of an isoleucine. Whether this serine residue is important for Aci01347‐mediated carnitine transport will be subject of future studies.

Three‐dimensional structures of the glycine betaine BCCT BetP of *C. glutamicum* revealed a long hydrophilic C‐terminal extension. This hydrophilic C‐terminal extension which is suggested to protrude into the cytoplasm were found to be crucial for activation of BetP by osmotic stress (Morbach & Krämer, 2002; Peter, Burkovski, & Krämer, 1998; Tøndervik & Strøm, 2007; Ziegler et al., 2010). Aci01347 exhibits only a short C‐terminal hydrophilic stretch of 53 hydrophilic residues which corresponds to the absence of osmoactivation of Aci01347.

We confirmed that *A. baumannii* uses carnitine as sole carbon and energy source. This together with the abundance of carnitine in the human host (Meadows & Wargo, 2015) suggests that carnitine is important for metabolic adaptation of *A. baumannii* to the human host. However, this might only be one physiological benefit of carnitine for *A. baumannii*. In *A. baumannii,* carnitine was found to be converted by the Rieske‐type oxygenase (CntAB) to trimethylamine (TMA; Zhu et al., 2014). The later is subsequently oxidized in the liver to the proatherogenic species trimethyl‐*N*‐oxide (TMAO) which correlates with human cardiovascular health (Koeth et al., 2013). It is tempting to speculate that carnitine uptake and subsequent degradation do not only play a role in metabolic adaptation to the human host but might also facilitate virulence. Whether carnitine metabolism of *A. baumannii* indeed plays a role in virulence will be subject of future studies.

## CONFLICT OF INTEREST

The authors declare no conflict of interest.

## AUTHORS CONTRIBUTION

JB, IW, and BA participated in design of the research, analysis of the data, and creating the manuscript. JB performed the experiments. All authors read and approved the final manuscript.

## ETHICS STATEMENT

This research did not involve studies with human or animal subjects, materials or data; therefore, no ethics approval is required.

## Data Availability

All data are included in the main manuscript or available as Supporting Information.
