# Supplementary material for: Identification and characterization of a carnitine transporter in Acinetobacter baumannii
Source: Microbiologyopen. 2018 Oct 14;8(6):e00752. doi: 10.1002/mbo3.752 (PMC6562126; doi:10.1002/mbo3.752)
Supplement: Supplementary file 1 [file MBO3-8-e00752-s001.doc]

**Suppl. 1:** **Primers used in the study**

| **Primer name** | **Sequence 5’ 🡪 3’** | **Application** |
| --- | --- | --- |
| pBAD/HisA_*aci01347*_fwd | AGCTGAGCTCCGCATGGATATGGATAATCAAAA | a |
| pBAD/HisA_*aci01347*_rev | GGCGGAATTCCGCTCTAGCTGGTTTTTCTATCC |
| *aci01347*_up_fwd | ATTAGCGGCCGCGTTTGCCATCTTCTGTCAGGCTAACG | b |
| *aci01347*_up_rev | CGCAGGATCCCACTGCATCCATGTTGCAGCAATC |
| *aci01347*_down_fwd | GCATGGATCCGTGACGGCTTTACCATTCATCGTG |
| *aci01347*_down_rev | ATTACTGCAGAAAGTTCATGGCCTCGGTGCGGACATAC |
| *aci01347*_ctr_fwd | ATCCATACGACCCGTAGGCGTACTGAG |
| *aci01347*_ctr_rev | TACGTGCCAATGCCAAGCTTCCATC |
| *aci01347*_compl_fwd | CATGGGATCCCGCCATCCAAAAATGGATGT | c |
| *aci01347*_compl_rev | CATGCTGCAGCTAGCTGGTTTTTCTATCCAG |
| *aci01347*_promotor_fwd | CATGGCGGCCGCTCAATTCATCTGCAACATCCC |
| *aci01347*_promotor_rev | CATGGGATCCTCACACCTTCGACTAATATCC |

^a^ Heterologous expression of *aci01347* in *E. coli* MKH13

^b^ Deletion of *aci01347* in *A. baumannii* ATCC 19606

^c^ Complementation of Δ*aci01347* with *aci01347*
